# Characterization of the interaction between platelet factor 4 and homogeneous synthetic low molecular weight heparins

**DOI:** 10.1111/jth.14657

**Published:** 2019-10-20

**Authors:** Thi-Huong Nguyen, Yongmei Xu, Sven Brandt, Martin Mandelkow, Ricarda Raschke, Ulrike Strobel, Mihaela Delcea, Wen Zhou, Jian Liu, Andreas Greinacher

**Affiliations:** 1 institut für Immunologie und Transfusionsmedizin, Universitätsmedizin Greifswald, Greifswald, Germany; 2 Institute for Bioprocessing and Analytical Measurement Techniques, Heiligenstadt, Germany; 3 ZIK HIKE—Center for Innovation Competence, Humoral Immune Reactions in Cardiovascular Diseases, University Greifswald, Greifswald, Germany; 4 Division of Chemical Biology and Medicinal Chemistry, School of Pharmacy, University of North Carolina, Eshelman, Chapel Hill, NC, USA; 5 Institute of Biochemistry, University Greifswald, Greifswald, Germany

**Keywords:** anti-PF4/heparin antibodies, interaction, length, platelet factor 4, synthetic heparins

## Abstract

**Background:**

Heparins are usually produced from animal tissues. It is now possible to synthesize heparins. This provides the abilities to overcome shortages of heparin, to optimize biological effects, and to reduce adverse drug effects. Heparins interact with platelet factor 4 (PF4), which can induce an immune response causing thrombocytopenia. This side effect is called heparin-induced thrombocytopenia (HIT). We characterized the interaction of PF4 and HIT antibodies with oligosaccharides of 6-, 8-, 10-, and 12-mer size and a hypersulfated 12-mer (S12-mer).

**Methods:**

We utilized multiple methodologies including isothermal calorimetry, circular dichroism spectroscopy, single molecule force spectroscopy (SMFS), enzyme immunosorbent assay (EIA), and platelet aggregation test to characterize the interaction of synthetic heparin analogs with PF4 and anti-PF4/heparin antibodies.

**Results:**

The synthetic heparin-like compounds display stronger binding characteristics to PF4 than animal-derived heparins of corresponding lengths. Upon complexation with PF4, 6-mer and S12-mer heparins showed much lower enthalpy, induced less conformational changes in PF4, and interacted with weaker forces than 8-, 10-, and 12-mer heparins. Anti-PF4/heparin antibodies bind more weakly to complexes formed between PF4 and heparins ≤ 8-mer than with complexes formed between PF4 and heparins ≥ 10-mer. Addition of one sulfate group to the 12-mer resulted in a S12-mer, which showed substantial changes in its binding characteristics to PF4.

**Conclusions:**

We provide a template for characterizing interactions of newly developed heparin-based anticoagulant drugs with proteins, especially PF4 and the resulting potential antigenicity.

## 1 | INTRODUCTION

Heparin is a sulfated and linear polysaccharide with a repeating disaccharide unit, containing iduronic acids and glucosamine residues.^1–3^ Each residue carries sulfate groups that influence the biological activities of heparin.^4^ Heparin is involved in many important biological processes^5^ such as viral/bacterial infection,^6,7^ angiogenesis,^8,9^ inflammation,^10,11^ and cancer metastasis.^12^ Pharmacologic heparin is an important drug for blood anticoagulation and an essential drug according to the World Health Organization.^13^

Heparin is purified from animal tissues, primarily porcine intestines,^5^ and heparins of different lengths are available including unfractionated heparin (UFH), different low molecular weight heparins (LMWH), and ultra-low weight heparins fractionated from UFH. The shortest anticoagulant heparin-like molecule, fondaparinux, a 5-mer resembling the binding site for antithrombin, is currently the only commercially available synthetic analog of heparin.^14,15^ In recent years it became possible to synthesize heparins of different lengths and to substitute these heparins by additional side groups.^16,17^ Such synthetic heparins will likely be the only way to overcome shortages of heparin in case of increasing demand. In addition, modification of the heparin chains might help to optimize biological effects or to overcome adverse effects. While animal-derived heparins are usually a polydisperse mixture of molecules, synthetic heparin analogs are homogeneous oligosaccharides. Each compound has a single sulfated carbohydrate sequence. The purity analysis and structural characterization of all these compounds have been reported.^18^

Treatment of patients with heparin can be associated with major adverse effects. Bleeding is the most prominent adverse effect, second are immune reactions toward complexes of heparin with proteins, especially platelet factor 4 (PF4). PF4 is a positively charged chemokine of the CXC family (also called CXCL4), which binds charge-related to polyanions including heparin. This can induce the production of antibodies, which recognize a neoepitope on PF4 in PF4/polyanion complexes. These antibodies can cause the adverse drug effect heparin-induced thrombocytopenia (HIT). HIT is a life-threatening, prothrombotic disorder in which antibodies binding to PF4/heparin (PF4/H) complexes activate platelets and monocytes resulting in increased thrombin generation. Previously, our group investigated the binding strengths between PF4 and animal-derived heparins.^19^ We found that PF4/H complexes are antigenic (ie, have the ability to bind to anti-PF4/H antibodies) if (a) the amount of anti-parallel β-sheets in PF4 exceeds ~ 30%, (b) multimolecular complexes are formed, and (c) the enthalpy of binding exceeds a threshold value of ~ 7 kcal/mol.^20^ We have also explored the binding pathways in PF4/H complexes, eg, short heparins bind to one-, while long heparins bind to two-PF4 tetramers.^21^ These interactions between PF4 and long heparins are a composite of charge-related binding and hydrophobicity among PF4 tetramers, resulting in stable PF4/H complexes.^22^

**FIGURE 1:**
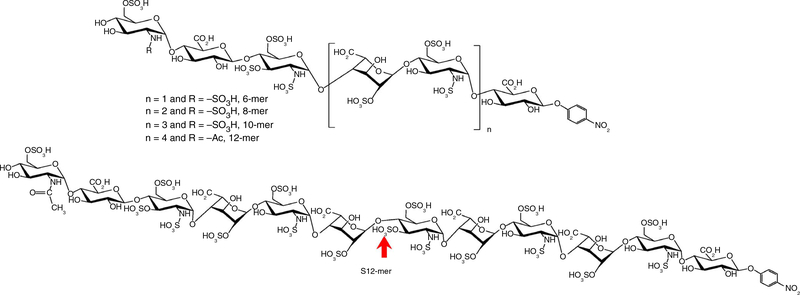
Chemical structures of homogeneous synthetic heparin oligosaccharides. Synthetic heparin-like compounds of 6-, 8-, 10-, and 12-mer correspond to n = 1; n = 2; n = 3; and n = 4, respectively (top panel). Super12-mer (S12-mer) was formed by adding an additional 3-*O*-sulfo group (indicated by the red arrow in the lower panel)

In this study, we characterized the interaction of synthesized heparins of different length including 6-, 8-, 10-, 12-mers, and a hypersulfated 12-mer (S12-mer) (Figure 1) with PF4 by different methods. The major advance in this study is to employ homogeneous oligosaccharides to study the binding to PF4 to eliminate the potential confounding effects introduced by different mixtures of oligosaccharides.

## 2 | METHODS

### 2.1 | Ethics

The use of platelets obtained from healthy volunteers and of anonymized sera obtained from patients with HIT was approved by the ethics board at the University of Greifswald.

### 2.2 | Synthesis of homogeneous heparin oligosaccharides

The synthesis of 6-mer, 8-mer, 10-mer, 12-mer, and S12-mer oligosaccharides was accomplished using a chemoenzymatic method. Purity and structural characterization of these oligosaccharides were presented previously.^18^ The detailed method of synthesis is described in Appendix S1 in supporting information. For testing of the 12-mer and S12-mer an aldehyde group was linked at one side of the heparins for subsequent experiments.

### 2.3 | Thermodynamics of PF4/heparin interaction by isothermal calorimetry (ITC)

The different heparins including 6-, 8-, 10-, 12-, and S12-mer and PF4 were separately dialyzed against PBS buffer at pH 7.4. ITC measurements were carried out using an iTC200 calorimeter (GE Healthcare Life Sciences) as previously described.^20^ Details are given in Appendix S1.

### 2.4 | Circular dichroism (CD) spectroscopy

Changes in the secondary structure of PF4 upon interaction with synthetic heparins 6-, 8-, 10-, 12-, and S12-mer were studied by recording far UV CD spectra (200–260 nm) using a Chirascan CD spectrometer (Applied Photophysics, Leatherhead, UK) as previously described.^23,24^ Details are given in Appendix S1.

### 2.5 | Enzyme immunoassay (EIA)

EIA was performed as described.^25^ First, the complexes of 20 μg/mL PF4 with heparins of different concentrations (ie, 1 to 10 μg/mL) were prepared in the fluid phase. Heparins of 6-, 8-, 10-, 12-, and S12-mer were used. Briefly, PF4/H complexes were immobilized on a microtiter plate to allow binding of aPF4/H Abs. For binding of aPF4/H Abs, each human serum was diluted (1:200) and incubated with PF4/H complexes coated on EIA plates for 1 hour at room temperature. Bound antibodies were subsequently detected by secondary goat anti-human IgG antibodies using a chromogenic substrate at wavelength 450 nm.

### 2.6 | Heparin-induced platelet activation assay (HIPA)

HIPA was performed as described.^26^ In brief, 75 μL of washed platelets were incubated with 20 μL sera with either the low molecular weight heparin (reviparin) 0.2 aFXaU or S12-mer (2.5 μg/mL) and lag time until platelet aggregation occurred measured every 5 minutes. A short lag time indicates strong platelet activation.

### 2.7 | Single-molecule force spectroscopy (SMFS)

PF4 was immobilized on 24 mm round glass coverslips mounted with gold and atomic force microscopy (AFM) tips were functionalized with the heparins as described.^22,27^ Details are given in Appendix S1.

## 3 | RESULTS

### 3.1 | Thermodynamics and secondary structure of PF4 upon complexation with heparins

**FIGURE 2:**
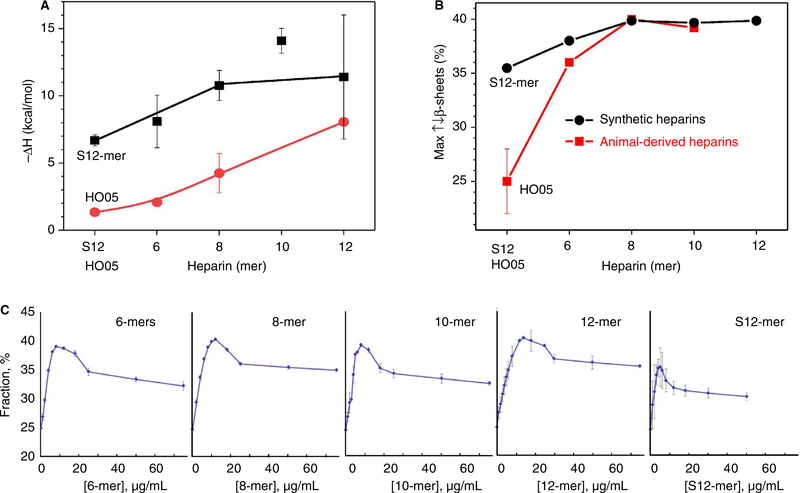
Thermodynamics and secondary structure of PF4 after interacting with heparins. (A) Enthalpy (ΔH) from the interaction of synthetic heparins (A, black) with PF4 increases and saturates at ≥ 8-mer. Enthalpy of the interaction between synthetic heparins and PF4 is always higher than that of the (A, red) animal-derived heparins. Enthalpy of the reaction between S12-mer was lower compared to other synthesized heparins tested. (B) Maximal ↑↓β-sheet content of PF4 in complex with synthetic heparins is highest for complexes formed with synthetic 8-, 10-, and 12-mers (B, black); it is lower for complexes formed with the 6-mer, and lowest for complexes formed with the S12-mer. Consistent with our previous findings,^20^ ↑↓β-sheet content of PF4 in its complexes with the animal-derived heparins (B, red) is highest for complexes with heparins ≥ 8-mer, lower for complexes with the 6-mer. The synthetic 5-mer fondaparinux induced by far the lowest ↑↓β-sheet content in PF4. (C) All compounds show a maximal conformational change at ~ 10 μg/ml. Long heparins ≥ 8-mer induced ~ 40% β-sheet content in PF4 structure while short heparin 6-mer or S12-mer induced < 40% β-sheet content. n = 3 repetition for each heparin. Note: Small error bars do not appear in the graphs

We first utilized ITC and CD spectroscopy to determine changes in enthalpy (ΔH) and secondary structure of PF4 after interacting with heparins. As ITC and CD spectroscopy do not require immobilization of PF4 or heparin, optimal interaction of freely orientated PF4 and heparin molecules in three dimensions could be obtained. The change in enthalpy when PF4 and heparin interact, as measured by ITC (Figure 2A, black), was negative, suggesting that the interaction of heparin and PF4 is an exothermic event. Larger negative values of ΔH were measured for complexes formed between PF4 and 8-, 10-, and 12-mer heparins, compared to complexes formed between PF4 and 6-mer heparin. This is expected because longer heparin chains can bring two PF4 molecules so close together that their charge clouds merge, which releases additional energy.^22^ Compared to complexes formed between animal-derived heparins or fondaparinux (HO05) with PF4 (Figure 2A, red), synthetic heparins in complex with PF4 released more heat. The most likely explanation is that animal-derived heparins are still polydisperse after fractionation. An alternative explanation might be that synthetic heparins may exhibit a higher degree of negative charges than the animal-derived heparins. Surprisingly, hypersulfated 12 (S12)-mer showed the lowest enthalpy among the synthesized heparins, although it exposes the most sulfate groups. However, the heat released by S12-mer was still in the same range as the heat released when animal-derived 12-mer heparin interacted with PF4. Table S1 in supporting information shows thermodynamic parameters for the interactions of PF4 with heparins.

The release of heat was accompanied by changes in the secondary structure of PF4 including random coil, antiparallel β-sheet (↑↓β-sheet) contents, β-turn, α-helix, and ↑↑β-sheet as measured by CD spectroscopy. Among these, we found the clearest change in ↑↓β-sheet contents, which we use in the following as a marker for the conformational changes. Animal-derived heparins at a certain length (Figure 2B, red) induced similar degrees of conformational changes in PF4 as the corresponding length synthetic heparins (Figure 2B, black), ie, highest for heparins ≥ 8-mer, lower for 6-mer, and lowest for the 5-mer fondaparinux (HO05). Conformational changes in PF4 when interacting with heparins of different concentrations are shown in Figure 2C. S12-mer induced also fewer changes in the conformation of PF4 than the other heparins but still substantially more than fondaparinux. Both enthalpy and conformational changes in PF4 saturated after interacting with heparins ≥ 8-mer, indicating different binding mechanisms among the investigated heparins with a boundary at 8-mer. Consistent with ITC, the S12- and 6-mer heparins induced lower ↑↓β-sheet contents in PF4 than the 8-mer, 10-mer, and 12-mer heparin (Figure 2B, black).

### 3.2 | Binding forces between synthetic heparins and PF4

**FIGURE 3:**
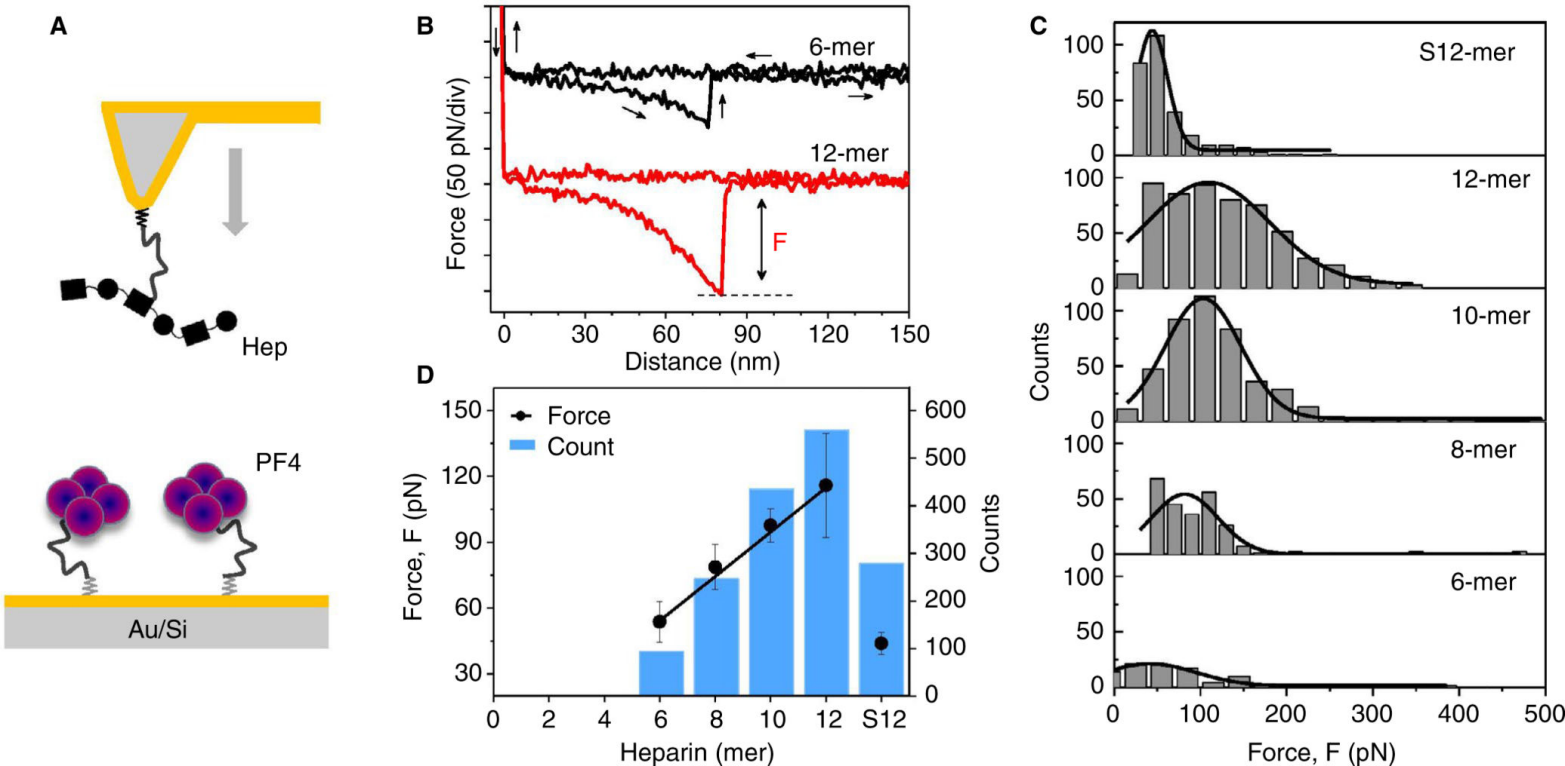
Binding strength between synthetic heparins of different length and PF4 on the solid phase. (A) 6-, 8-, 10-, 12-mer heparins and S12-mer heparin had been covalently bound to the tips of the cantilever, while PF4s were covalently linked to the Au-surface, both via PEG linkers. When the tip approaches the substrate, heparin interacts with PF4 and when it moves away from the substrate, the rupture force is recorded. (B) Typical force-distance curves measured with synthetic 6-mer (black) and 12-mer (red) heparins showing rupture forces *F* which are collected in (C) histogram distributions. (D) Mean values and the associated standard errors show a linear dependence of binding force versus heparin length. Surprisingly the S12-mer bound with much weaker forces (black) and with lower frequencies of interaction or count (blue)

To gain insights into the interaction of synthetic heparins of different length with PF4, we determined the binding force between PF4 immobilized on the substrate and different synthetic heparins linked to atomic force microscopy (AFM) tips by SMFS-based atomic force microscopy (Figure 3A). Long polyethylene glycol (PEG) spacers (~30 nm) between the tip and the heparin molecule or between the substrate and PF4 were utilized as described.^22^ Force-distance (F-D) curves showing stretching behavior of a polymer were selected for analysis. As an example, higher rupture forces were obtained for the interaction of PF4 with 12-mer heparin (red curve in Figure 3B) compared to the interaction of PF4 with 6-mer heparin (black curve in Figure 3B).

Histogram distributions of rupture forces F between PF4s and synthetic heparins of different length are given in Figure 3C. The synthetic 10- and 12-mer heparins interacted with PF4s much more strongly than the synthetic 6-, 8-, and surprisingly also S12-mer (Figure 3C). Average binding force and frequency of interaction (counts) increased with increasing heparin length, with the exception of the S12-mer (Figure 3D). The results indicate that synthetic heparins 10- and 12-mer form more bonds with PF4 than 6- and 8- and S12-mer heparins.

### 3.3 | Antigenicity of PF4/synthetic heparin complexes tested by enzyme immunoassay (EIA)

**FIGURE 4:**
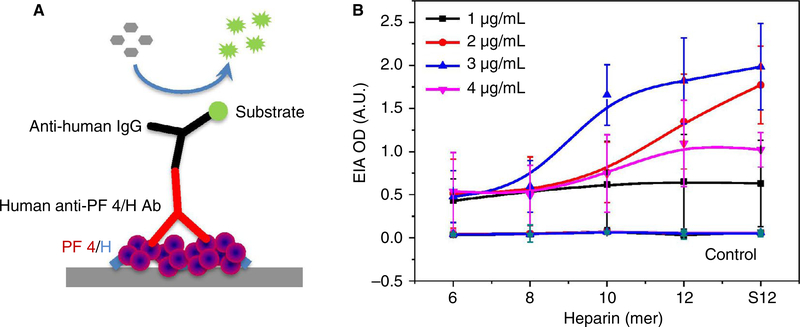
Binding of aPF4/H Abs to PF4/H complexes in EIA. (A) A substrate coated with PF4/H complexes was incubated with human sera known to contain aPF4/H Abs. Binding of human antibodies to PF4/H complexes was then detected by a labeled secondary goat antihuman IgG antibody (= antihuman IgG Ab). Binding of aPF4/H Abs was quantified by the change in optical density (OD) which is proportional to the amount of substrate activated by the labeled secondary antibody. (B) EIA OD (five independent sera/each data point) for antibody binding to complexes formed between PF4 (20 μg/mL) with various concentrations of different synthetic heparins shows an increase in OD for 10-mer, 12-mer, and S12-mer heparins but no significant change for 6-mer and 8-mer heparins. Averages of three sera without aPF4/H Abs served as controls and showed ODs < 0.5 at all heparin concentrations

Antigenicity means that the PF4/synthetic heparin complexes expose a neoepitope on PF4, which is induced by the conformational change, allowing aPF4/H Abs to bind. EIA plates were coated with preformed complexes between PF4 and synthetic heparins of different length and binding of aPF4/H Abs was detected by a labeled secondary goat anti-human IgG antibody (Figure 4A). Antibody binding was quantified by the change in optical density (OD). When forming complexes at the same PF4 concentration with heparin concentrations ranging from 1 to 10 μg/mL, OD increased with increasing concentrations for synthetic 10-mer, 12-mer, and S12-mer heparins, while no significant change in OD occurred for heparins ≤ 8-mer (Figure 4B, bottom line). Detailed results for each heparin are shown in Figure S1 in supporting information. As controls, three sera without aPF4/H Abs were tested in parallel with all heparins at different concentrations (Figure 4B). These controls showed no binding of antibodies (OD < cutoff of 0.5) regardless of heparin lengths and concentrations. The above results are consistent with the findings of ITC and CD spectroscopy with the exception of S12-mer heparin. Among synthetic heparin compounds, the S12-mer heparin interacted with PF4 most weakly in the liquid phase but in complex with PF4, it allowed strong binding of aPF4/H Abs.

### 3.4 | Characteristics of S12-mer heparin in the functional heparin-induced platelet activation (HIPA) test

**FIGURE 5:**
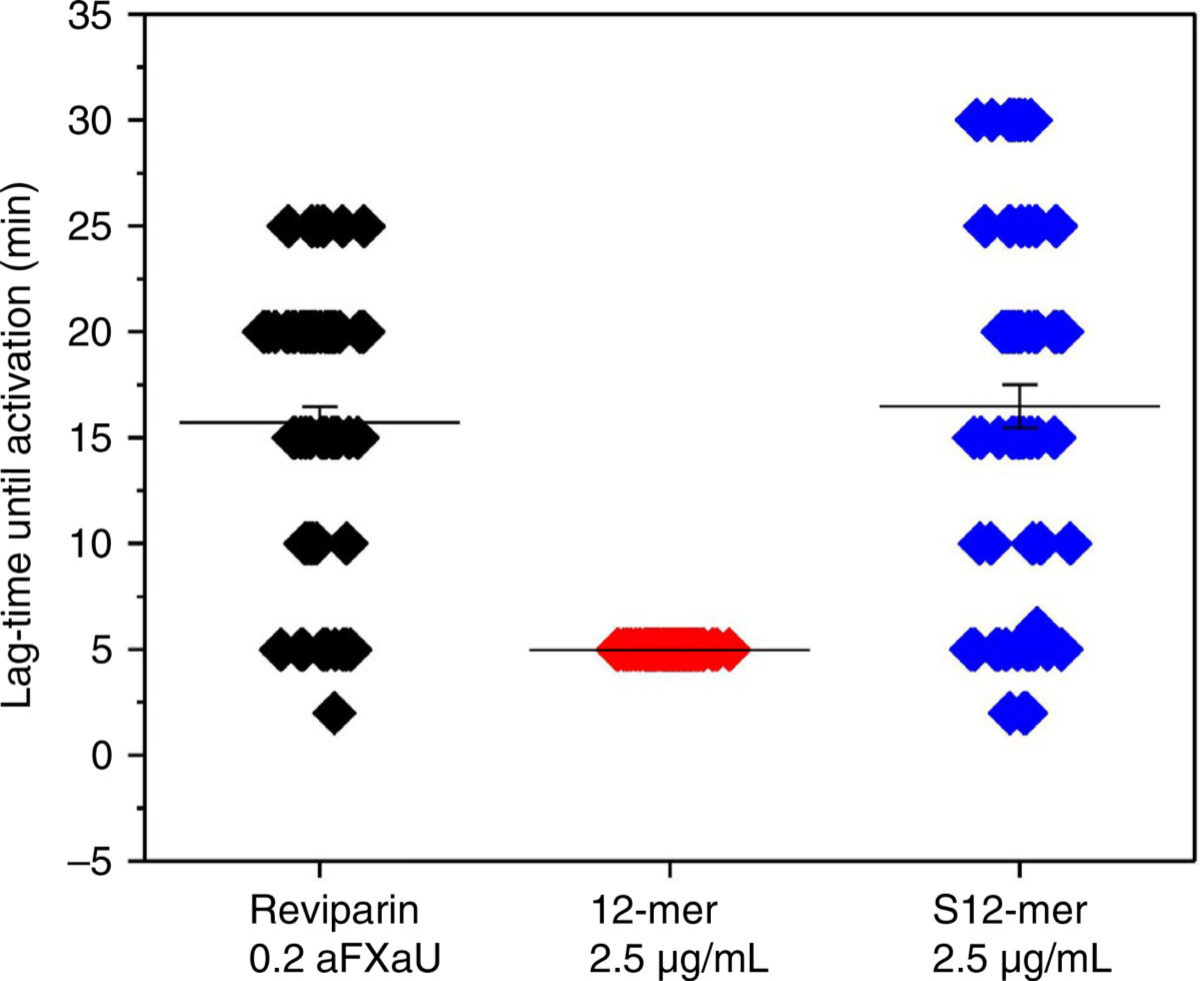
S12-mer tested in HIPA. Anti-PF4/H antibodies induced similar platelet activation in the presence of both S12-mer (blue) and the standard animal-derived LMWH reviparin (black) but much more weakly as compared with the 12-mer. The lag time is a marker for the strength of platelet activation. Results of testing 70 sera containing anti-PF4/heparin antibodies are shown. Min = minutes; aFXa = anti-Factor Xa units

When we incubated platelets of 74 independent donors with 15 sera known to contain platelet-activating antibodies with S12-mer heparin, in comparison with reviparin (a standard animal-derived LMWH), both induced platelet activation in the presence of the human aPF4/H Abs (Figure 5 and Figure S2 in supporting information) but at much longer lag time of platelet activation than 12-mer heparin (Figure 5, red). In the presence of normal control sera (without heparin-induced antibodies) neither reviparin nor S12-mer induced platelet activation. These results are consistent with the findings by EIA that the S12-mer induces exposure of the antigen on PF4 to which aPF4/H Abs bind, and are also compatible with the findings obtained by CD spectroscopy showing slightly reduced conformational changes of PF4 in the presence of S12-mer, which still result in> 30 % ↑↓β-sheets, and with a slightly lower enthalpy measured by ITC. It is, however, not consistent with the much lower binding forces of S12-mer to PF4 in SMFS.

### 3.5 | Evidence for different structures of 12- mer and S12-mer heparin

**FIGURE 6:**
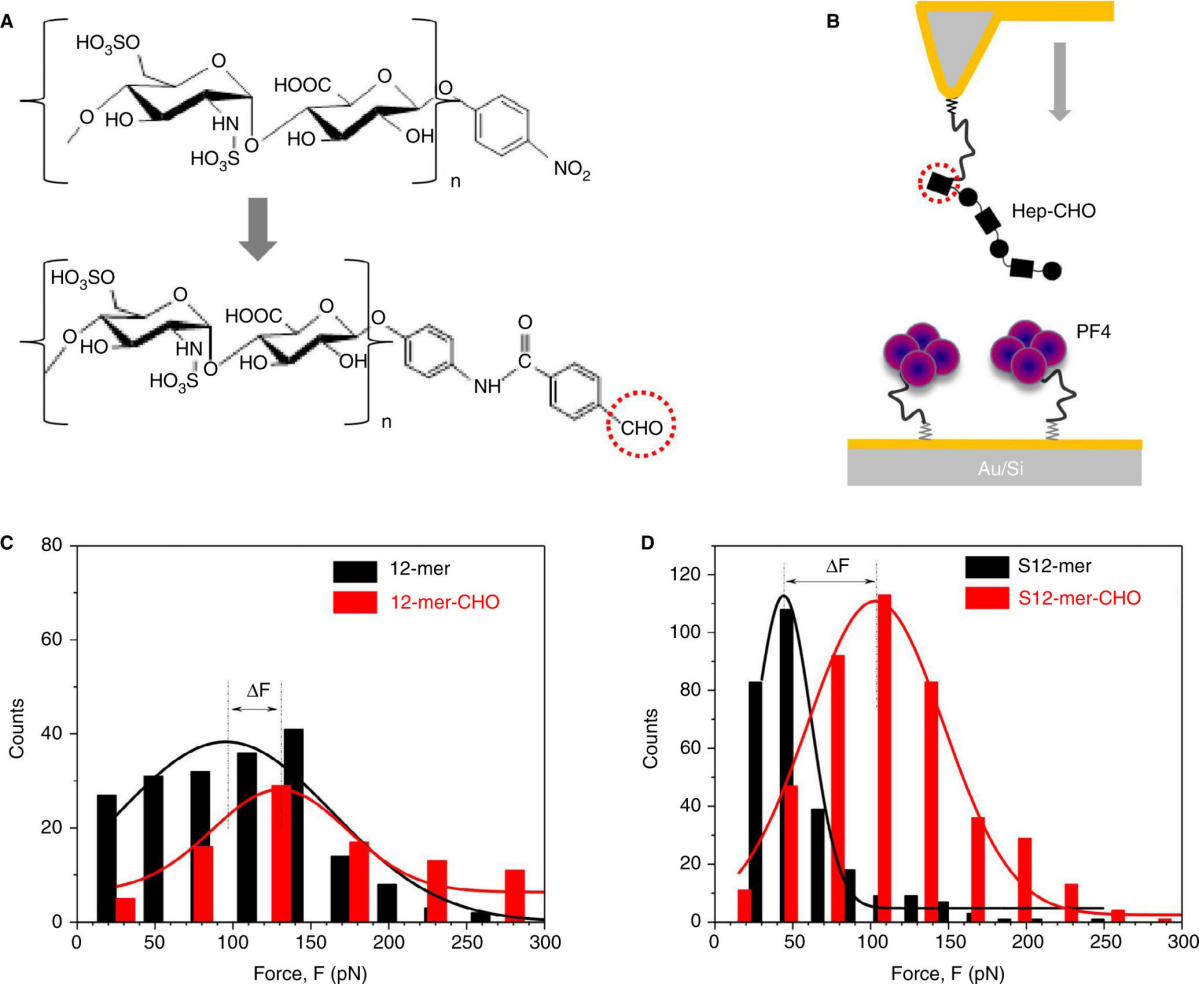
Enhancement of binding forces of 12-mer heparins with an additional functional -CHO group with PF4. (A) Chemical structures showing the aldehyde group introduced to one end of the 12- or S12-mer heparin (red circle). (B) Schematic of the experimental setup: an end of heparin is covalently immobilized on the AFM tip via a PEG linker (red circle), while the rest of the molecule freely orients and interacts with PF4 on the substrate (please compare with Figure 2A). For (C) 12-mer heparin and especially for (D) S12-mer heparin, the binding force of the randomly covalently immobilized heparins to the tip (black) is lower than that of the directed covalent bond at the end of the heparins (red) as indicated by the different binding forces (ΔF)

The discrepant findings for S12-mer obtained by EIA compared to the SMFS experiments may be due to the fact that the interaction of S12-mer and PF4 were impaired in the SMFS experiments. In the above SMFS experiments, we randomly activated carboxyl groups along the heparin chains to form covalent bonds with the PEG linker on the tip (Figure 3A). This results in binding of heparin chains to the tip at random sites. A heparin chain may fold differently depending on the carbohydrate involved in PEG-linker binding. This may impact the interaction with PF4. We, therefore, introduced a -CHO group at one end of 12-mer of S12-mer heparin (Figure 6A, red). This allows a directed covalent amide bond between the -CHO group of heparin and -NH_2_ group at the end of PEG on the AFM tip in the presence of NaCNBH_3_ (Figure 6B). With this design, 12-mer-CHO heparin showed slightly increased binding forces compared to randomly linked 12-mer as shown by a shift in force ΔF by ~ 35 pN (Figure 6C). However, binding forces of S12-mer-CHO strongly increased by ΔF ~ 60 pN (Figure 6D) but they are still weaker than the ones of 12-mer. When we compared the formation of PF4 complexes with heparins with and without CHO by CD spectroscopy and ITC, CHO-linked 12-mer and S12-mer showed similar conformational changes and heat release compared to 12-mer and S12-mer (Figure S3 in supporting information).

## 4 | DISCUSSION

By combining various analytical methods including CD spectroscopy, SMFS, ITC, EIA, and HIPA, we have characterized the interaction of synthesized heparins of various length with PF4 and their potential for antigenicity.

By SMFS, we observed that synthetic heparins of 6-, 8-, 10-, and 12-mer length bind more strongly to PF4 than animal-derived heparins of corresponding lengths. We also observed by CD spectroscopy and ITC that changes in ↑↓β-sheet contents and changes in enthalpy are higher when synthetic heparins complex with PF4 than those observed when animal-derived heparins form complexes with PF4. A likely explanation is that the animal-derived heparins are still polydisperse after fractionation, potentially containing some smaller fragments, which reduce the overall signal. Another potential reason could be that the synthetic heparins are slightly more sulfated than animal-derived heparins. Nevertheless, both heparin types bind with a similar trend, ie, weak binding for heparins ≤ 8-mer and strong binding for heparins ≥ 10-mer. When we performed interaction studies of PF4 with animal-derived heparins of different length we found a clear correlation between the conformational change in PF4 induced by these heparins, as measured by CD spectroscopy, and binding of aPF4/H antibodies to PF4/heparin complexes. In the present study, both ITC and CD spectroscopy gave similar results for synthetic 8-mers and synthetic 10-mers for −ΔH and antiparallel beta-sheet content, respectively. However, binding of aPF4/H antibodies to these complexes as measured by enzyme immunoassay differed substantially with nearly no reactivity between antibodies and PF4/8-mer complexes and strong reactivity between PF4/10-mer complexes (Figure S1). We have previously proposed that biophysical assays alone can predict the potential of negatively charged molecules to induce immunogenic changes in PF4.^20^ The present study confirms this for most tested synthesized heparins. However, it also shows that this might not be entirely true for molecules close to the critical lengths. The 8-mer and 10-mer synthesized heparins differ in length by two glucose moieties while they have otherwise identical structures. They behave similarly in the biophysical assays but showed differences in the biological assays. We, therefore, expand our previous recommendation to test for polyanions with a structure close to the borderline (which can be determined by biophysical assays) in addition to biophysical assays the reactivity of resulting complexes with aPF4/H antibodies as an additional parameter before the development of the compound is stopped due to fear of immunogenicity.

Due to the availability of new monoclonal antibodies mimicking human aPF4/H antibodies like KKO^28^ or 5B9^29^ such testing has become much easier for research laboratories.

A plausible explanation for the generally higher heat release in ITC and increased conformational change in PF4 in CD-spectroscopy of the synthetic heparins is that they are uniform; unfractionated animal-derived heparins may still contain smaller fragments.

Especially interesting are the interactions of the S12-mer heparin with PF4. It interacts with PF4 more weakly than does 12-mer. S12-mer heparin, which has a higher degree of negative charge than the original 12-mer, was expected to show stronger binding characteristics to PF4. However, compared with other synthesized heparins, S12-mer heparin induced lowest conformational changes in PF4 in CD spectroscopy experiments, and released the weakest heat in ITC tests, and bound to PF4 with lowest binding force. While this is potentially biologically relevant, it does not affect the risk of interacting with aPF4/H Abs. S12-mer still showed substantial antigenicity as shown by strong binding of human anti-PF4/H antibodies (ie, high OD values in EIA) and promoted activation of platelets in the presence of aPF4/H Abs similar to low molecular weight heparin. This is not too surprising as it still showed negative enthalpy (ΔH ~ 7 kcal/mol) and an increase of antiparallel beta-sheets in PF4 of about 35%.

Most interestingly, we observed a major difference in the characteristics of S12-mer binding to PF4, depending on whether S12-mer was linked randomly to the AFM-tip or when the end of this compound was covalently linked to the tip via a specific CHO group. The S12-mer-CHO bound site directed to the AFM tip showed much stronger binding forces to PF4 compared to the randomly bound S12-mer. This difference was not observed for the 12-mer-CHO compared to 12-mer heparin. This is not caused by different interaction between S12-mer-CHO and S12-mer with PF4, as both showed the same reactivity in ITC and CD spectroscopy in regard to heat release and antiparallel beta-sheet induction in the fluid phase (Figure S3). Rather, this indicates that the distinct bindings of PF4 to S12-mer and S12-mer-CHO may be due to the spatial structure of S12-mer. S12-mer is unique as it carries two 3-O-sulfated glucosamine residues that have a large impact on the conformation of IdoA2S residues. The IdoA2S residues present in S12-mer exit in both chair (^1^*C*_4_) and skew boat (^2^*S*_O_) conformers, and the conformation is governed by the sulfation patterns.^30^ The 3-O-sulfation has been reported to drive the nearby IdoA2S toward ^2^*S*_O_ conformation.^31^ It is possible that the spatial orientation of the sulfo groups dictates the binding affinity to PF4. The orientation of the sulfo groups is determined by the conformation of IdoA2S residues. As we observed stronger binding between S12-mer and PF4 when the oligosaccharide is affixed on the AFM tip through the reducing end, this is consistent with the hypothesis that such presentation provides greater structural flexibility of S12-mer to interact with PF4 and to wrap around the ring of positive charges of PF4 to reach the state of lowest energy.^32,33^ This dependency on the binding site of the PEG linker is not the case for 12-mer heparin. This strongly indicates that the 12-mer heparin is likely less folded compared to S12-mer and binding of the PEG-linker either to the CHO-end or to random binding sites to the 12-mer chain had no major impact on the binding forces. This experiment with S12-mer-CHO provides indirect evidence that even minor changes in heparins may have major effects on their structure. This may have a large impact on their interaction with other molecules and potentially also on their biological activities.

Carbohydrate-based drugs are considered to have a major effect on the development of new pharmacological compounds.^34^ Our study shows how newly developed heparin-like drugs can be characterized for their potential antigenicity. It also shows that each individual carbohydrate may require a special experimental design to fully understand its characteristics.

## Supplementary Material

supplementary materials

## Essentials

- Synthetic heparin-like compounds display stronger binding characteristics to PF4 than the animal-derived heparins.
- Minor modifications of synthetic heparin-like compounds may result in changes in the safety profile of these drugs.
