# Supplementary material for: Characterization of the interaction between platelet factor 4 and homogeneous synthetic low molecular weight heparins
Source: J Thromb Haemost. Author manuscript; Available in PMC 2021 Feb 1. (PMC7236814; doi:10.1111/jth.14657)
Supplement: supplementary materials [file NIHMS1592020-supplement-supplementary_materials.docx]

**Supplementary Information**

Characterization of the Interaction between Platelet Factor 4 and Homogeneous Synthetic Low Molecular Weight Heparins

Thi-Huong Nguyen,^1,2,3*^ Yongmei Xu,^5^ Sven Brandt,^3^ Martin Mandelkow,^3^ Ricarda Raschke,^1^ Ulrike Strobel,^1^ Mihaela Delcea,^3,4^ Wen Zhou,^5^ Jian Liu^5^ and Andreas Greinacher^1*^

*^1^Institute for Immunology and Transfusion Medicine, University Medicine Greifswald, 17475 Greifswald, Germany.*

*^2^Institute for Bioprocessing and Analytical Measurement Techniques, Heiligenstadt, Germany*

*^3^ZIK HIKE - Center for Innovation Competence, Humoral Immune Reactions in Cardiovascular Diseases, University Greifswald, 17489 Greifswald, Germany.*

*^4^Institute of Biochemistry, University Greifswald, 17489 Greifswald, Germany.*

*^5^Division of Chemical Biology and Medicinal Chemistry, Eshelman, School of Pharmacy, University of North Carolina, Chapel Hill,* NC 27599, USA.

**Supplementary Fig. 1.** B**inding of anti-PF4/H Abs in EIA.** Anti-PF4/H Abs bind to complexes formed between PF4 and 6- and 8-mer heparin much weaker than those formed by heparins ≥10-mer, suggesting only 6- and 8-mer may be non-antigenic.

Supplementary Fig. 2. Reactivity of reviparin *vs* S12-mer tested in HIPA. Each point represents one serum tested with reviparin (black) or S12-mer (red).

**Supplementary Fig. 3: Thermodynamics and secondary structure of PF4 after interacting with12-mer and 12-mer CHO and S12-mer and S12-mer-CHO.** Both 12-mer and S12-mer do not show a significant difference in (**A**) enthalpy (ΔH) or (**B, C**) maximal ↑↓ ß-sheet content of PF4.

Supplementary Table 1: Thermodynamic parameters for the interaction of PF4 with the synthetic heparin.

|  | |  |  |  |  |
| --- | --- | --- | --- | --- | --- |
| **Synthetic heparin** | | | **Animal-derived heparin** | |  |
| **Heparin (mer)** | Stoichiometry, N | K_A_ x 10^6^ [M^-1^] | **Heparin (mer)** | Stoichiometry, N | K_A_ x 10^6^ [M^-1^] |
| **S12** | 0.2 | 8.0 |  | ------- | ------ |
| **12** | 2.3 | 5.0 | ***HO12*** | 1.1 | 3.2 |
| **10** | 1.2 | 0.3 | ***HO10*** | **------** | **------** |
| **8** | 1.2 | 0.4 | ***HO08*** | 1.9 | 5.0 |
| **6** | 1.3 | 5.0 | ***HO06*** | 0.4 | 0.4 |
| **5** | **------** | **------** | ***HO05*** | 1.3 | 0.8 |

**Methods**

**Synthesis of homogeneous heparin oligosaccharides**

The synthesis of 6-mer, 8-mer, 10-mer, 12-mer, and S12-mer oligosaccharides was accomplished using a chemoenzymatic method. The purity and structural characterization of these oligosaccharides were presented in a previously published publication [1]. Briefly, heparosan synthase-2 (PmHS2) from Pasteurella multocida was used to elongate the monosaccharide, GlcA pNP, to the appropriate sized backbone. The backbone was then subjected to the modification by N-sulfotransferase (NST), C5-epimerase (C5-epi), 2-O-sulfotransferase (2-OST), 6-O-sulfotransferase (6-OST), 3-O-sulfotransferase isoform 1 (3-OST-1), and 3-O-sulfotransferase isoform 5 (3-OST-5). Five major steps were involved in the overall synthesis, including elongation to add GlcNTFA or GlcA), Detrifluoroacetylation/N-sulfation, 2-O sulfation/epimerization, 6-O-sulfation, 3-O-sulfation by 3-OST-1 or 3-OST-5. Elongation step was to elongate the oligosaccharide backbone to the desired size, involving the addition of GlcNTFA or GlcA residue. For example, GlcA-pNP (3.2 mM) was dissolved in buffer containing Tris (25 mM, pH 7.2), MnCl2 (5 mM), pmHS2 (60 µg/ml), and UDP-GlcNTFA (4.5 mM), then incubated at 30°C overnight. A C18-column (3 × 15 cm, or 120 g, Biotage) was used for purification with gradient elution method (0-100% methanol in H2O, 0.1% trifluoroacetic acid, 5 ml/min). Step of detrifluoroacetylation and N-sulfation were aimed to convert a GlcNTFA residue to a GlcNS residue, involving both detrifluoroacetylation and N-sulfation. The detrifluoroacetylation of the oligosaccharide was conducted in 0.1 M LiOH (pH >12.0) under an ice bath for 0.5 h. The product formation was monitored by electrospray ionization mass spectrometry (ESI-MS). After the reaction completed, pH was immediately adjusted to 7.0 using hydrochloric acid (1 M). For example, the detrifluoroacetylated tetrasaccharide (1.3 mM, GlcNH2-GlcA-GlcNH2-GlcA-pNP) was N-sulfated in a solution containing 2-(Nmorpholino) ethanesulfonic acid (MES, 50 mM), pH 7.0, and N-sulfotransferase (32 μg/ml) and PAPS (1.5 molar equivalent of free amino groups) at 37°C overnight in a reaction volume of 5.6 L.

Step of epimerase and 2-OST was to convert a GlcA residue to an IdoA2S residue using both C5-epi and 2-OST. For example, the oligosaccharide GlcNTFA-GlcA-GlcNS-GlcA-GlcNS-GlcA-pNP (1.2 mM) was incubated in a solution containing Tris (25 mM) buffer (pH 7.5) and semipurified C5-epi (3 μg/ml), 2-OST (6.5 μg/ml), and PAPS (1.8 mM) at 37°C overnight in a reaction volume of 4.9 L. 6-O-sulfation was to introduce 6-O-sulfation using both 6-OST-1 and 6-OST-3. The oligosaccharide intermediate (0.5 mM) was incubated in a buffer containing MES

(50 mM, pH 7.0), 6-OST-1 (50 μg/ml), 6-OST-3 (50 μg/ml), and PAPS (1.5 molar equivalent of 6-hydroxyl groups) at 37°C overnight in 1 L. 3-O-sulfation was to introduce 3-O sulfation by 3-OST. The oligosaccharides (0.5 mM) were incubated in a solution containing MES (50 mM) buffer (pH 7.0), 3-OST-1 or 3-OST-5 (20 μg/ml), and PAPS (0.675 mM) in 0.9 L at 37°C overnight. All the sulfated products were purified using Q-Sepharose fast flow column (GE Life Science) eluted with a linear gradient 0-100% 2 M NaCl in 20 mM NaOAc-HAcO, pH 5.0 for 3h. Different sizes of Q-Sepharose columns were chosen based on the binding affinity of the product and the reaction scale. At every synthesis step before the 6-O- and 3-O-sulfation step, the products were monitored by Shimadzu HPLC equipped with a polyamine II column (4.6 mm x 250 mm, from YMC). After the steps of 6-O-sulfation and 3-O-sulfation, the products were monitored by a DEAE-NPR column (4.6 mm x 75 mm, from Tosoh Bioscience). The MWs of the intermediates from each step was confirmed by electrospray ionization mass spectrometry (ESI-MS).

**Heparin with aldehyde group**

To a solution of synthetic heparin 12-mer or S12-mer (100 µg) dissolved in 1mL of dd water a catalytic amount of 10% Pd/C was added, the mixture was stirred for 2h at the atmosphere of H_2_, after removal of Pd/C using high-speed centrifugation, the crude residue 12-mer or S12-mer -NH_2_ was obtained for next reaction without further purification.

The crude residue 12-mer or S12-mer -NH_2_ was re-dissolved in 1mL of 50 mM (pH=5.0) NaAc buffering solution and 1,4-dioxane (V/V=1:1), and then excessive amount of 2,5-dioxopyrrolidin-1-yl 4-formylbenzoate was added, and the mixture was stirred overnight at r.m., and ultimately the resulting mixture was purified with NPR column HPLC (TSK gel. DNA-NPR, 4.6mm x 7.5 cm x 2.5 µM) to give the target compound 12-mer or S12-mer-CHO (Supplementary Fig. 4).

Supplementary Fig. 4. Chemistry used to introduce CHO group to heparin end.

**Thermodynamics of PF4/Heparin interaction by Isothermal Calorimetry (ITC)**

The different heparins including 6-, 8-, 10-, 12-, and S12-mer and PF4 were separately dialyzed against PBS buffer at pH 7.4. ITC measurements were carried out using an iTC200 calorimeter (GE Healthcare Life Sciences). A PF4 solution (180 µM in PBS) was added to the sample cell, and a solution of heparin (10 µM) was loaded into the injection syringe. For each experiment, a 60-second delay was followed by 19 injections of 1 µL of the titrant solution, spaced 240 seconds apart. The sample cell was stirred at 1,000 rpm throughout and maintained at 25 °C. Control titration was performed by injecting heparin into PBS buffer and subtracted prior to data analysis. The area under each peak of the resulting heat profile was integrated, normalized by the concentrations, and plotted against the molar ratio of heparin to PF4 using an Origin script supplied with the instrument (Origin 7; OriginLab Corporation). The resulting binding isotherms were fitted by nonlinear regression using the single-site model. The stoichiometry of the interaction (N = C_Heparin_/C_PF4_, where C is the concentration in moles/liter), the equilibrium constant (K_A_) and the change in enthalpy (ΔH) were obtained during the fitting of all titration data. All titrations were replicated to determine the experimental standard deviation for each parameter.

**Circular dichroism (CD) spectroscopy**

Changes in the secondary structure of PF4 upon interaction with synthetic heparins 6-, 8-, 10-, 12-, and S12-mer were studied by recording far UV CD spectra (200–260 nm) using a Chirascan CD spectrometer (Applied Photophysics, Leatherhead, UK) like previously described [2]. PF4 was dissolved in phosphate-buffered saline (PBS; 155 mM NaCl, 1.54 mM KH_2_PO_4_, 2.71 mM Na_2_HPO_4_-7H_2_O, pH 7.2) to final concentrations of 80 µg/mL. Complex formation was carried out at 20°C directly within the CD cuvette (Hellma, Müllheim, Germany). Each measurement started with a pure PF4 solution whose initial concentration was set to 80 µg/mL (cuvette path length = 5 mm). Afterward, increasing amounts of the 6-, 8-, 10-, 12-, and S12-mer heparins were sequentially added to the cuvette (leading to defined PF4/H mixtures) and a CD spectrum was recorded for each mixing step. Additionally, buffer baselines and baselines of each heparin concentration step (without PF4 in the solution) were recorded. In the data analyses, the spectra of PF4 alone and of PF4/H complexes were corrected for the baselines, path length and concentration to obtain the wavelength dependent mean residue delta epsilon (MRDE) values of the PF4/H complex. To estimate the secondary structure content of PF4, deconvolution of CD-spectra was carried out with CDNN (software, circular dichroism neural network) using a database of 33 reference proteins. In the deconvolution, process calculations were adjusted for the moderate dilution of PF4 (due to the addition of the heparin solution).

**Enzyme immunoassay (EIA)**

EIA was performed as described [3]. First, the complexes of 20 µg/ml PF4 with heparins of different concentrations (i.e. 1 to 10 µg/ml) were prepared in the fluid phase. Heparins of 6-, 8-, 10-, 12-, and S12-mer were used. Briefly, PF4/H complexes were immobilized on a microtiter plate to allow binding of aPF4/H Abs. For binding of aPF4/H Abs, each human serum was diluted (1:200) and incubated with PF4/H complexes coated on EIA plates for 1h at RT. Bound antibodies were subsequently detected by secondary goat anti-human IgG antibodies using a chromogenic substrate at wavelength 450 nm.

**Heparin-induced platelet activation assay (HIPA)**

HIPA was performed as described [4]. In brief, 75 µL of washed platelets were incubated with 20 µL sera with either the low molecular weight heparin (reviparin) 0.2 aFX aU or S12-mer of 20 µg/ml.

**Single molecule force spectroscopy (SMFS)**

***Immobilization of PF4***

24 mm round glass coverslips (Plano GmbH, Wetzlar, Germany) were cleaned using RCA standard cleaning procedure (a 1:1:5 solution of NH_4_OH:H_2_O_2_:H_2_O; 10 min at 70°C), dried under nitrogen stream and exposed to oxygen plasma (600W, oxygen flow 500 sccm, Gigabatch 310, PVA TePla, Wettenberg, Germany) for 10 min. Subsequently, the freshly cleaned glass coverslips were sputter coated (Q150R S, Quorum Technologies, East Grinstead, United Kingdom) with chromium and then with gold (Au). Subsequently, the gold-coated surface was rinsed with acetone, ethanol, and ultrapure water and dried under nitrogen stream before exposing to oxygen plasma for 30 min to remove organic components. Next, the surfaces were immersed into a solution of 1 mg/mL alpha-thio-omega-carboxy poly(ethylene glycol) (HS-PEG-COOH, PEG 3000 Da, Iris Biotech GmbH, Marktredwitz, Germany) in ethanol for 2h at room temperature (RT) to functionalize the gold surfaces with PEG-COOH linkers [5-7]. The coated substrates were then rinsed five times with deionized water to remove unbound molecules. After drying under a nitrogen stream, the carboxyl groups at the end of PEG linkers were activated for 1h in the amine coupling kit (Biocore, Uppsala, Sweden) containing the mixture of 0.4 M 1-ethyl-3-(3-dimethylaminopropyl) carbodiimide hydrochloride (EDC) and 0.1 M *N*-hydroxysuccinimide (NHS). The substrates were then rinsed thoroughly with water and incubated at RT for 1h in 100 µg/mL platelet factor 4 in PBS (PF4, Chromatec, Greifswald, Germany) [8]. Finally, the samples were rinsed three times with PBS and used within a day.

***Functionalization of AFM tip with heparin***

Both sides gold-coated silicon nitride cantilevers with nominal spring constant of 6 pN/nm (Olympus Biolever, Tokyo, Japan), were exposed under UV-ozone cleaner for 30 min. The cantilevers were then incubated in 1 mg/mL thiol-PEG-amine in ethanol (HS-PEG-NH_2_, PEG MW 3400 Da, Nanocs, USA) for 2h and rinsed five times with PBS [9]. The carboxyl groups along synthetic heparins (6-, 8-, 10-, 12-, and S12-mer) in water were activated by with EDC:NHS kit (as described above) for 1h, at RT (Supplementary Fig. 5A-B). Afterward, the cantilevers were incubated in the heparin solution for 1 h at RT and finally rinsed with water [6]. The cantilevers were used for SMFS measurements within a day.

SMFS measurements were carried out in PBS using JPK NanoWizard 3 (Berlin, Germany). Before any experiment, the cantilever spring constant was independently measured by a thermal tune procedure [10, 11]*.* To compare the change in the binding force among heparins, force-distance (F-D) curves were recorded at the same tip velocities (1,000 nm/s). A 200 pN setpoint was used to control the maximal force of the tip against the surface. For all investigated heparins, the measurements were repeated at least three times using independently prepared cantilevers and freshly PF4 coated surfaces. Typically, for each heparin, 1,000 F-D curves were recorded. The rupture forces and rupture distances were extracted from the F-D curves analyzed using the JPK data processing software (version 4.4.18+). The mean rupture force values and their corresponding errors were determined by applying Gaussian fits to the data using Origin software (version 8.6).

To form covalent bond between -CHO group at the end of heparins to the tip, the cantilever was coated with HS-PEG-NH_2_ ethanol (MW 3400 Da, Nanocs, USA). Heparin-CHO was incubated with the cantilever-PEG-NH_2_ in the presence of 1M NaCNBH_3_ 1h, RT (Supplementary Fig. 5C). After rinsing, the cantilever was ready to be used.

Supplementary Fig. 5. Chemistry used to form covalent bonds with AFM tips. (A) AFM tip is functionalized with PEG-NH_2_ *via* Au-S bond. (B) Carboxyl groups on heparin chains are activated by EDC:NHS before covalent amine bonds between PEG and heparins are formed. (C) Aldehyde group at the end of heparins are activated prior to formation of covalent bond with PEG on the tip.

**References**

1 Xu YM, Cai C, Chandarajoti K, Hsieh PH, Li LY, Pham TQ, Sparkenbaugh EM, Sheng JZ, Key NS, Pawlinski R, Harris EN, Linhardt RJ, Liu J. Homogeneous low-molecular-weight heparins with reversible anticoagulant activity. *Nat Chem Biol*. 2014; **10**: 248-+.

2 Brandt S, Krauel K, Gottschalk K, E., Renne, Thomas, Helm C, A., Greinacher A, Block S. CD-Spectroscopic Assessment of potential Antigenicity Induced by Negatively Charged Drugs. *ACS Nano*. 2013.

3 Juhl D, Eichler P, Lubenow N, Strobel U, Wessel A, Greinacher A. Incidence and clinical significance of anti-PF4/heparin antibodies of the IgG, IgM, and IgA class in 755 consecutive patient samples referred for diagnostic testing for heparin-induced thrombocytopenia. *Eur J Haematol*. 2006; **76**: 420-6.

4 Greinacher A, Amiral J, Dummel V, Vissac A, Kiefel V, Muellereckhardt C. Laboratory Diagnosis of Heparin-Associated Thrombocytopenia and Comparison of Platelet-Aggregation Test, Heparin-Induced Platelet Activation Test, and Platelet Factor-4 Heparin Enzyme-Linked-Immunosorbent-Assay. *Transfusion*. 1994; **34**: 381-5.

5 Wildling L, Unterauer B, Zhu R, Rupprecht A, Haselgrubler T, Rankl C, Ebner A, Vater D, Pollheimer P, Pohl EE, Hinterdorfer P, Gruber HJ. Linking of Sensor Molecules with Amino Groups to Amino-Functionalized AFM Tips. *Bioconjugate Chem*. 2011; **22**: 1239-48.

6 Nguyen TH, Lee SM, Na K, Yang S, Kim J, Yoon ES. An improved measurement of dsDNA elasticity using AFM. *Nanotechnology*. 2010; **21**: 075101(1)-(7). Artn 075101

7 Takano H, Kenseth JR, Wong SS, O'Brien JC, Porter MD. Chemical and biochemical analysis using scanning force microscopy. *Chem Rev*. 1999; **99**: 2845-90.

8 Nguyen TH, Greinacher A. Platelet factor 4/heparin complexes present their epitopes differently on a solid phase system than on the platelet surface. *Blood*. 2017.

9 Nguyen TH, Steinbock LJ, Butt HJ, Helm M, Berger R. Measuring Single Small Molecule Binding via Rupture Forces of a Split Aptamer. *J Am Chem Soc*. 2011; **133**: 2025-7.

10 Hutter JL, Bechhoefer J. Calibration of Atomic-Force Microscope Tips. *Rev Sci Instrum*. 1993; **64**: 1868-73.

11 Butt HJ, Jaschke M. Calculation of Thermal Noise in Atomic-Force Microscopy. *Nanotechnology*. 1995; **6**: 1-7.
